# Dynamic guardianship of potato landraces by Andean communities and the genebank of the International Potato Center

**DOI:** 10.1186/s43170-021-00065-4

**Published:** 2021-11-27

**Authors:** Sophia Lüttringhaus, Willy Pradel, Víctor Suarez, Norma C. Manrique-Carpintero, Noelle L. Anglin, David Ellis, Guy Hareau, Nelissa Jamora, Melinda Smale, Rene Gómez

**Affiliations:** 1 Genebank Impacts Fellow, CGIAR Genebank Platform, Platz der Vereinten Nationen 7, 53113 Bonn, Germany; 2 Sustainable Land Use and Climate Change, Department of Agricultural Economics, Humboldt University of Berlin, Unter den Linden 6, 10117 Berlin, Germany; 3 Member of the Leibniz Association, Potsdam Institute for Climate Impact Research (PIK), Telegrafenberg, 14473 Potsdam, Germany; 4 Social and Health Sciences and Innovation Systems. International Potato Center, Av. La Universidad 1895, La Molina, Lima 12, Peru; 5 International Potato Center, Program for Conserving Biodiversity for the Future, Av. La Universidad 1895, La Molina, Lima 12, Peru; 6 USDA ARS Small Grains and Potato Germplasm Research, Pacific West Area, 1691 S. 2700 W., Aberdeen, ID 83210 USA; 7 Global Crop Diversity Trust (Crop Trust), Platz der Vereinten Nationen 7, 53113 Bonn, Germany; 8 Michigan State University, 446 W. Circle Dr., Rm 219, Justin S Morrill Hall of Agriculture, East Lansing, MI 48824-1039 USA

**Keywords:** Peru, Potato landraces, Repatriation program, International Potato Center (CIP), Genebank, Food security, Household survey, Duration analysis, Survival, Benefits

## Abstract

**Background:**

Potato landraces (*Solanum *spp.) are not only crucial for food security and sustenance in Andean communities but are also deeply rooted in the local culture. The crop originated in the Andes, and while a great diversity of potato persists, some landraces have been lost. Local communities and the genebank of the International Potato Center (CIP) partnered to re-establish some of these landraces in situ by supplying clean seed potatoes to farmers. Over time, the genebank formalized a repatriation program of potato landraces. Repatriation is the process of returning native germplasm back to its place of origin, allowing a dynamic exchange between ex situ and in situ conditions. So far, no comprehensive description of CIP’s repatriation program, the changes it induced, nor its benefits, has been carried out.

**Methods:**

We addressed this research gap by analyzing CIP genebank distribution data for repatriated accessions, conducting structured interviews with experts of the repatriation program, and applying duration and benefit analyses to a survey dataset of 301 households.

**Results:**

Between 1997 and 2020, 14,950 samples, representing 1519 accessions, were distributed to 135 communities in Peru. While most households (56%) abandoned the repatriated material by the fourth year after receiving it, the in situ survival probability of the remaining material stabilized between 36% in year 5 and 18% in year 15. Households where the plot manager was over 60 years old were more likely to grow the repatriated landraces for longer periods of times. While male plot management decreased survival times compared to female plot management, higher levels of education, labor force, wealth, food insecurity, and geographic location in the southern part of Peru were associated with greater survival times. Most farmers reported nutritional and cultural benefits as reasons for maintaining landrace material. Repatriated potatoes enabled farmers to conserve potato diversity, and hence, re-establish and broaden culinary diversity and traditions.

**Conclusions:**

Our study is the first to apply an economic model to analyze the duration of in situ landrace cultivation by custodian farmers. We provide an evidence base that describes the vast scope of the program and its benefits.

## Background

In the Andes, potato landraces (*Solanum *spp.) are strongly embedded in the local culture and therefore constitute the backbone of food security and livelihoods. The crop originated in the Andes, where a great diversity developed by natural and human selection still exists today (Parra-Rondinel et al. 2021). It is estimated that about 4000 varieties of native potatoes or landraces are cultivated in the Andes, 3000 are present in Peru (Parra-Rondinel et al. 2021). Landraces of potato are locally adapted genotypes that are conserved by farmers in heterogeneous Andean conditions (Arce et al. 2019; Parra-Rondinel et al. 2021). Under these conditions, landrace diversity is crucial to create resilient production systems that ensure farmers’ food security (Bellon 1996; Brush 2004; Burgos et al. 2009; de Haan 2009; Jackson et al. 2012). However, over time, smallholder farming communities have lost some landraces due to susceptibility to biotic and abiotic stresses, climate change, terrorism (Ellis et al. 2020), changes in the marketability or cultivation system and personal preferences (de Haan, 2009). Consequently, in the 1990s, the idea to return lost landraces to their places of origin evolved from the long-term partnership between the genebank at the International Potato Center (CIP) and the Peruvian smallholder farming communities in the Andes. These redistribution activities are summarized under the designation of a “repatriation program”, which has the direct intention to improve farmers’ food security and to increase the infraspecific potato diversity managed on-farm amidst the local knowledge, traditions, and landscapes in which the material evolved. Over time activities have expanded and have become a part of routine genebank work at CIP. While called a “program”, however, repatriation activities have never received dedicated funding or other resources.

According to CIP staff and participating farmers, the repatriation program provides many benefits to the receiving communities, particularly culinary and cultural benefits (R. Gómez, personal communication, 13 October 2020, Ellis et al. 2020), but to date, neither the program, nor the benefits and changes it induces have been properly documented or comprehensively analyzed. When generally referring to on-farm conservation projects, Bellon et al. (2015) noted that the empirical evidence for their effectiveness is often insufficient. To overcome this research gap and contribute to the literature about on-farm conservation of landraces, we first developed a comprehensive description of the repatriation activities to date, drawing on the passport data of repatriated landraces, and the expertise and experience of CIP staff directly involved in this effort. Second, we applied a duration model to identify household and community factors that influence the survival of the repatriated landraces on farms or in situ. Finally, we used a benefit and change analysis to investigate the changes brought about by the repatriation program to Andean smallholder farmer communities and their individual members. This work is the first systematic description of the implementation, outcomes, and impacts of the potato repatriation program for participating farmers since it began 24 years ago. CIP’s repatriation activities are described, and evidence is documented, including the timespan farmers utilize the repatriated materials.

The term “repatriation” is generally used to describe the redistribution of material from international or foreign genebanks to national genebanks. In the CIP context, it also refers to the reintroduction of landraces from the CIP genebank back to their places of origin in the Peruvian Andes for direct on-farm use and in situ or on farm maintenance (R. Gómez, personal communication, 13 October 2020). In 1997, the first repatriation effort by CIP started when a group of farmers participating in a local potato fair contacted the genebank to help them quantify the level of available potato diversity in their communities (R. Gómez, personal communication, 24 August 2020). Within this context, the idea of redistributing healthy landrace germplasm back to the places of origin for in situ conservation evolved dynamically through dialogues between CIP staff and Andean communities (Huaman et al. 2000). In 1999, the term “repatriation” was used for the first time in an internal CIP report (Huaman 1999). Besides the repatriation program, the CIP genebank also processes individual national distribution requests of native germplasm to clients including farmers. Normally, that germplasm is distributed as in vitro plantlets, but those distributions are not part of the scope of this paper.

CIP’s repatriation process embodies a dynamic and circular model of ex situ and in situ conservation. During this process valuable landraces are returned to Andean communities (exchanged from ex situ to in situ conditions), and in exchange novel landraces identified from these communities are deposited into the genebank for long-term conservation and safe keeping (exchange from in situ to ex situ). In the latter case, the landraces fill genetic gaps in the potato germplasm collection at CIP and add value to the germplasm collection. Such a dynamic model of ex situ and in situ conservation has a long intellectual legacy developed by Bellon et al. (1997), Berthaud (1997), Maxted et al. (1997), Ortega (1997), McLean-Rodríguez et al. (2019) and Ocampo-Giraldo et al. (2020).

**Fig. 1:**
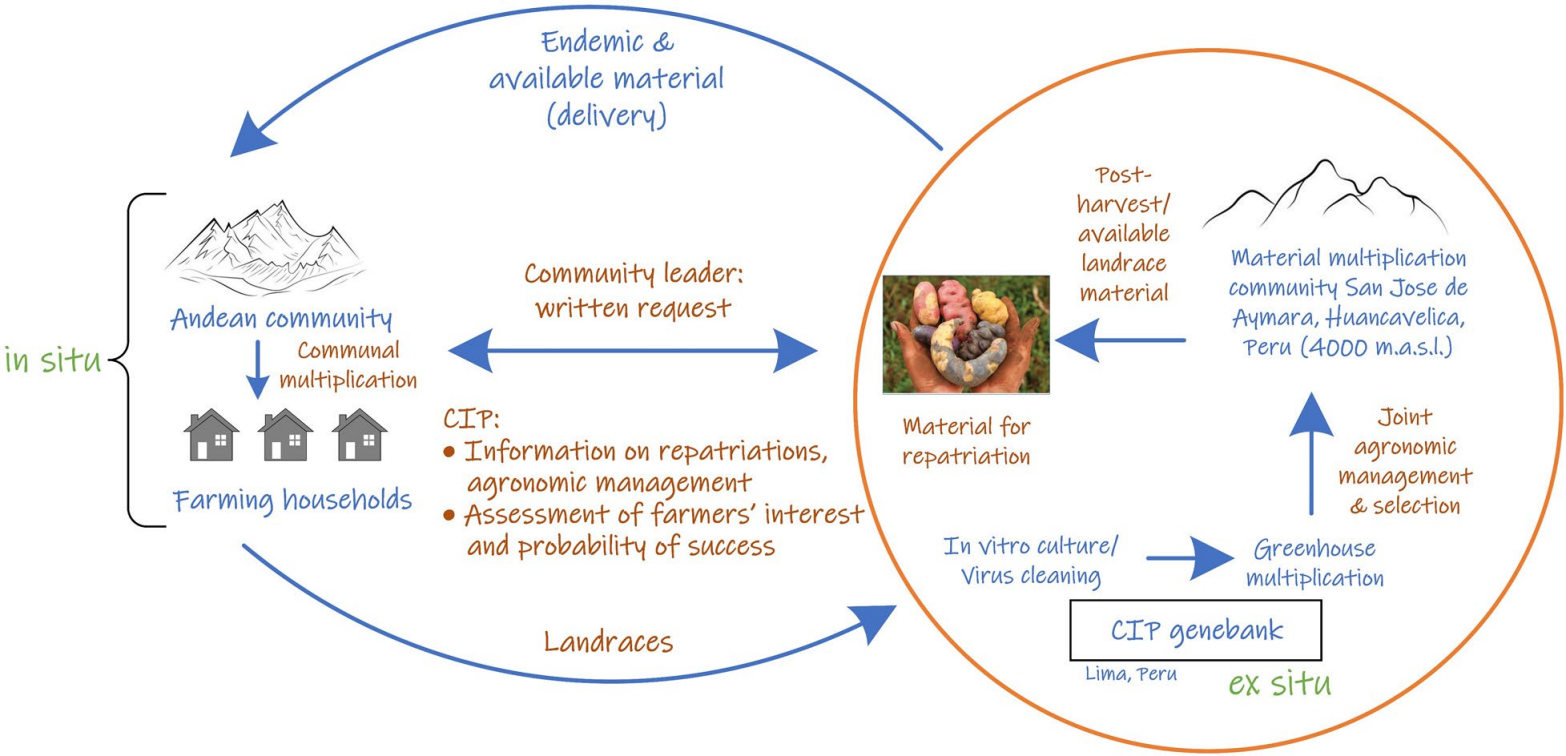
Scheme of the repatriation program. The left, blue circle displays the mutual material exchange between the CIP genebank and Andean communities, and the orange circle on the right illustrates the material conservation, cleaning, and multiplication process to generate material for repatriation

The processes and stakeholders of CIP’s repatriation program are mapped out in Fig. 1. There is a continuous cycle of exchange between ex situ and in situ conservation activities, which are inherently linked to one another, while some activities occur simultaneously. This link and dynamic exchange likely only occurred due to the building of personal relationships between the Andean farmers and genebank staff, which in turn encouraged farmers to entrust their landraces to be deposited back to the genebank. When deposited in the genebank the landraces are transferred with the Standard Material Transfer Agreement (SMTA) from the International Treaty on Plant Genetic Resources for Food and Agriculture (ITPGRFA) and thus become a global public good available and accessible for requestors for research, education, and breeding. All material is transferred with an SMTA—in both directions. As a public good, material can be supplied back to farmers in case they were to lose these landraces, and farmers can use them according to paragraph 9.3 of the ITPGRFA. The graph and workflow can be divided into two parts: (1) the large circle on the left-hand-side, which describes the exchange between the CIP genebank and the participating Andean communities and (2) the smaller orange circle, which represents the tuber multiplication and is a prerequisite to redistribute material. The tuber multiplication for the CIP genebank is carried out every year with the support of the community of San José de Aymara, located in the Andean highlands. Members of this community are contracted annually and renumerated to multiply genebank potato accessions, a continuous routine genebank activity to maintain viable accessions. Hence, the community plays a central role in ensuring that sufficient healthy tubers are available for repatriation and further genebank use.

The repatriation process begins with a formal written request from a community authority or head to CIP. This request is then reviewed by those continually involved in the repatriation program who check which accessions are suitable and available for repatriation to a specific location. Selection is made based on two criteria: availability (1) and suitability (2) of the material. The first criterion is the most important as this is the bottleneck regarding repatriation. Availability is determined by the multiplication planning of the genebank and production constraints in San José de Aymara. This means that the repatriation team decides on a best fit between all available material and its suitability for the communities requesting repatriation. When the accession or landrace tuber’s origin is from the region of a requesting community a good suitability is implied. Hence, endemic potatoes are frequently redistributed back to their places of origin which means that most landraces are strongly associated with the cultural heritage of the receiving communities and will grow well in that area. Additionally, other material with a broader geographic distribution range can also be repatriated to a community when the repatriation team deems it suitable according to their experience and the specific request(s) made by the community such as for specific traits, tastes and textures.

Nevertheless, it is important to note that the specific landrace selection is not completely demand-driven as availability is the binding criterion. In most cases, communities request a repatriation without stating specific preferences e.g., on traits or growing requirements and they cannot select the specific landraces they will receive since the CIP genebank contains thousands of landraces derived from Peru. When the communities state specific preferences, they are accommodated if suitable material is available. After this selection, usually the applying communities receive samples of 8–10 tubers per landrace (R. Gómez, personal communication, 01 March 2021). After receiving the tubers, the communities organize the communal tuber multiplication and the corresponding division of labor and plots. When the community has produced enough tubers, the landraces are redistributed to individual farming households for inclusion in their own potato-planting portfolios. The lower large arrow denotes the flow of material between in situ and ex situ conservation. Here it is important to note that the reciprocal exchange is indispensable for the repatriation program to ensure longevity and safe backup of these landraces. Since certain potato varieties have been grown in their place of origin for over 8000 years, the Andean landscapes and farmers, have conserved the landraces over time and also generated new diversity (Hawkes 1988; Popenoe et al. 1989).

The smaller circle describes the flow of repatriation work that is necessary to prepare (multiply) the material in the genebank for redistribution back to the Andean farmers. The genebank prepares the material by planting in vitro generated plantlets in greenhouses to produce mini tubers. The mini tubers are then used for planting in potato plots to finally produce the tubers for repatriation. The final multiplication occurs jointly with the community of San José de Aymara, located at about 4000 m above sea level (m.a.s.l.) in the province of Huancavelica in Peru. The high altitude of the community ensures less biotic stressors for the regenerated material, and hence, facilitates the growth of healthy material for distribution (Quiroz et al. 2018). At the end of the multiplication process, the most suitable and promising landrace cultivars are selected, processed, and checked for visible disease, true-to-type, health, etc. The available material for repatriation marks the starting point of the big circle, where the genebank sends the clean material to participating Andean communities. In some cases, farmers approach CIP with specific requests for specific landraces or tastes, textures, colors, and other traits in mind, and then the principal curator tries to best match these wishes based on the farmers description with the available material. The annual costs for the repatriation program at CIP are estimated at below US$ 5000 (D. Ellis and N. Anglin, personal communication, 10 March 2021). This cost is low as the production of clean tubers is a routine genebank activity (for accessions maintenance, characterization, taxonomic determination, genotyping, etc.) and thus tubers are available for other uses, such as repatriation.

## Data and methods

Two main data sources were employed in this study: (1) accession-level genebank distribution data on all repatriated landraces from 1997 to 2020 and (2) household survey data that were collected in August and September 2018 with the purpose of investigating and better understanding the changes and benefits resulting from the repatriations.

The CIP genebank maintains distribution data on the repatriated landraces and receiving communities. This data includes accession number, Global Information System Digital Object Identifier (GLIS-DOI), cultivar name, taxon name, geographic origin, collecting site, genebank accession status, requestor details (name, location, reason for request), and some morphological traits. On the community level, the data contains the names and locations of the receiving communities, their reasons for participating in the repatriation, the year when the repatriation was carried out and which landraces they received. The data set covers the complete time span of the repatriation program starting in 1997 and for the analyses presented here, all data from 1997 to 2020 was used.

The household survey was conducted by CIP with the goal to create an evidence base of the changes induced by the repatriation program and to document how the repatriated landraces were used by the farmers. For this survey, 301 households and community leaders who had participated in the repatriation program were interviewed in 65 communities, covering half of all the communities that received repatriations over the history of this program (CIP 2018). Households were interviewed in the South and Center of Peru, based primarily on logistical reasons (ease of reaching the communities). As the survey covers half of all communities that had participated in the program until September 2018 and also includes the important potato diversity hotspots, the household survey was thus considered to be representative of repatriation recipients. The survey was comprehensive and contained 79 variables of different types, retrieving household characteristics and demographic data such as location, age, gender, education, labor force and wealth, information on the repatriation process and, potato landrace production in the communities, as well as perceived changes regarding food security, potato production, poverty reduction, self-identity. Also, the conservation of repatriated landraces was surveyed, including questions where farmers were asked to recall the year when they first and last planted repatriated material. We used the data from the genebank to summarize the outputs of the repatriation program and analyze the accession-level information on the repatriated landraces. Mixed methods were used for analysis, as numeric variables were aggregated, and text variables were encoded and clustered. To complement the information from the database, six semi-structured interviews were held with the principal potato curator at CIP, Rene Gómez, who has been the main coordinator for the repatriated accessions and principal CIP liaison for the repatriation program since its inception.

We employed survival or duration models, which originated in the field of medicine for analyzing the survival of patients after specific diseases and treatments (Kaplan and Meier 1958) and were later used in other disciplines, such as agricultural economics, to study the adoption of agricultural innovations, such as improved varieties (Fuglie and Kascak 2001; Burton et al. 2003; Dadi et al. 2004; Matuschke and Qaim 2009; Alcon et al. 2011; Oostendorp and Zaal 2012; Beyene and Kassie 2015; Nazli and Smale 2016; Ray and Maredia 2016; Lemessa et al. 2019; Ofori et al. 2020).

In econometrics, a duration model aims to study the expected time an individual spends in one state before transitioning to another, measured with a dichotomous variable (Alcon et al. 2011). In our case, this variable measured the number of years a farming household continued to grow a repatriated landrace after receiving it. We refer to this period as the survival time of the repatriated landrace in that household. A duration analysis describes the probability of an event happening (e.g., loss or abandonment of a landrace) at a given point in time (Beyene and Kassie 2015). The method can be classified as parametric, semi-parametric, and non-parametric. Parametric methods, such as binomial, Poisson, Weibull, or logistic distributions, have assumptions for the maximum likelihood or least squares estimators, which restrict their use when time is the key variable and data is censored (Cleves et al. 2016). Non-parametric methods, such as the Kaplan–Meier function (Kaplan and Meier 1958), analyze the success or failure of an event over time. With the Kaplan–Meier curve, we can demonstrate how high the probability of survival (i.e., a farmer continues to maintain a repatriated landrace) is at each point in time. Semi-parametric models, such as the Cox proportional-hazards model (Cox 1972), permit the use of explanatory variables (covariates) and interact with non-parametric methods to explain their results.

We used a duration model to predict how long farmers will cultivate repatriated landraces and which factors influence survival time. The survival time is a mark of success for the repatriation program because the longer farming households cultivate the repatriated landraces, the longer they may benefit from them. The duration model consisted of two parts. First, we generated a Kaplan–Meier survival curve to estimate the baseline survival times. Second, we used a Cox proportional-hazards model to estimate the survival time with several covariates which influence the probability of survival. This multivariate analysis showed which household or community characteristics influenced the survival time.

The Kaplan–Meier model describes how long an event continues (survival time) in the presence of censored data. In our case, censored items were farming households where the landraces still survived when the survey was conducted in 2018, but about whom we had no follow-up data that told us if the landraces still survived today. It is important to note that the surveyed farming households participated in different years in the program. The repatriation program started in 1997, and since then, many farmers received repatriated material each year. The sample included households in the sample who received material in 1997, but also those who had participated for only one year when being surveyed. The analysis integrated the censored items in such a manner that it did not distort the duration of the uncensored items.

The Kaplan–Meier is a maximum likelihood estimator, denominated $\widehat{S}\left(t\right)$:

1 $$\widehat{S}\left(t\right)= \prod\nolimits_{{i: t}_{i}<t}\frac{{n}_{i}-{d}_{i}}{{n}_{i}}$$

where ${t}_{i} is$ a point in time when at least one event happened, ${d}_{i} is$ the number of events that happened at time ${t}_{i}$, and ${n}_{i}$ are the individual households where the repatriated landraces are still maintained, i.e. they have not yet abandoned the material or been censored up to time ${t}_{i}$.

This model is univariate (time is the only variable), and it is characterized by the assumption that survival does not increase over time, all participants have the chance to change their status, and all respondents will change their status at some point in time. This function estimated the probability that landrace maintenance will continue based on observed times. The resulting stepwise function showed the probability that the repatriated landraces still survive in a farming household at a certain point in time.

As a second step, we conducted a Cox proportional-hazards model to calculate the probability of survival, given certain household or community characteristics and given that this household has maintained the landraces until a certain point in time. The Cox proportional-hazards model was calculated as:

2 $$\gamma \left(\tau , {X}_{1},\dots ., {X}_{n}\right)={\gamma }_{0} \left(\tau \right) exp \left({\sum }_{i=1}^{n}{\beta }_{i}{X}_{i}\right)$$

where ${\gamma }_{0} \left(\tau \right)$ is the base risk and corresponds to the risk of abandoning the landraces when all variables have zero value and the $exp({\sum }_{i=1}^{n}{\beta }_{i}{X}_{i})$ depends on the predictor variables or covariates. This multivariate analysis showed us which household or community characteristics influence the survival time to abandonment. The effect of each variable was given as the hazard ratio (HR). A HR > 1 indicated an increased likelihood of abandoning the repatriated material in situ as compared to the median value across all samples, and an HR < 1 indicated a decreased likelihood. Here the likelihood was defined as the probability of occurrence of the model event at each successive year after a household received repatriated material.

The incentives for smallholder farmers to grow landraces are not well understood. Brush et al. (1992) were the first researchers to investigate potato diversity on farms in the Andes. Hence, their work was an important basis for analyzing the in situ conservation enabled by the repatriation program. We are guided in our choice of hypothesized determinants (Table 1) by duration models of variety choice (Nazli and Smale 2016; Ray and Maredia 2016), other adoption models (Matuschke and Qaim 2009), and also crop diversity analyses derived from the non-separable model of the household farm (Smale 2006; Smale et al. 2001; Meng 1997; Van Dusen 2000), in which imperfect markets lead to landrace or variety choices that address both the production and consumption needs of the household. Thus, household characteristics such as labor supply and wealth are expected to influence the capacity of the household to undertake additional farm work. In particular, the characteristics of the plot manager affect access to new information and resources, such as planting material. Production and consumption are conditioned on factors measured at a higher scale of analysis, which we measure by district food insecurity and geographical zone. Abiotic stressors exacerbated by climate change have a large impact on Andean potato production. The food security index was established by WFP and CENEPRED (2015) and quantifies the probability that a population will be food insecure due to natural phenomena. Generally, the range of factors we were able to test was limited by the scope of the data.

**Table 1:** Variable selection for the Cox proportional model

Variable	Description	Summary statistics: mean (std. dev.) or frequency counts, NAs
*Time variable* Time	Survival time, number of years a community receives the repatriated landraces to the last year where farmers plant these varieties (time to abandonment)	4.29 (4.38) NAs = 43
*Event/status variable* Event	Dummy variable (1 = abandonment of repatriated landraces has happened, 0 = otherwise)	0 (n = 96) 1 (n = 172) NAs = 33
*Covariates/ Independent variables* Gender	Binary gender (male or female) of the person managing the plots with repatriated potatoes	Male (n = 246) Female (n = 49) NAs = 6
Age	Age group of the person managing the plots with repatriated potatoes (below 30 = plot manager is below 30 years of age, 30–60 = plot manager is between 30 and 60 years, 60 + = plot manager is older than 60)	Below 30 (n = 20) 30–60 (n = 205) 60 + (n = 65) NAs = 11
Education	Education of the person managing the plots with repatriated potatoes (none = 0, initial = 1, primary = 2, secondary = 3, technical = 4, tertiary = 5, other = 6)	0 (n = 14) 2 (n = 174) 3 (n = 84) 4 (n = 6) 5 (n = 11) NAs = 12
Labor force	Number of internal (household members) and external people who help with agricultural work	8.84 (5.16) NAs = 12
Wealth	Number of services (drinking water, drainage, electricity, telephone, TV, internet) per household	2.63 (1.33) NAs = 11
Food insecurity	Index of Peruvian food insecurity in the face of recurrent natural disasters. Average per district (index running from 0.00 (= no risk of food insecurity) to 0.85 (= very high risk of food insecurity)^a^	0.36 (0.22) NAs = 4
Zone	Geographical zone in Peru: Center includes the departments Ancash, Huánuco, Pasco, Lima, Junín, Huancavelica; South includes Ayacucho, Apurímac, Cusco, Arequipa, Puno^b^	Center (n = 123) South (n = 178)

^a^Sourced from WFP and CENEPRED (2015) ^b^For more information, see Fig. 2

Tests were carried out to confirm the suitability and robustness of the model. To avoid multicollinearity, the correlations among independent variables were checked and found not to be statistically significant. Model diagnostics were conducted, including a test on the Weibull distribution and a test of the proportional hazard assumption based on scaled Schoenfeld residuals. Additionally, the model results were tested for influential observations.

Finally, we analyzed the changes and benefits perceived by farmers based on the feedback from the household survey. This analysis further explained why farmers maintained and abandoned their repatriated material, and hence, provided valuable insights on how the repatriation program produced changes and how possible benefits can be enhanced and upscaled as the repatriation program continues. This information also enabled us to better understand the reasons for landrace abandonment by revealing foregone benefits. For the benefit and change analysis, multiple choice, and open-ended survey responses to relevant questions were analyzed descriptively. Therefore, all answers were categorized and then the number of farmers whose answer belonged to a certain category was counted.

## Results

### Genebank data analysis: characterizing the repatriated diversity

**Fig. 2:**
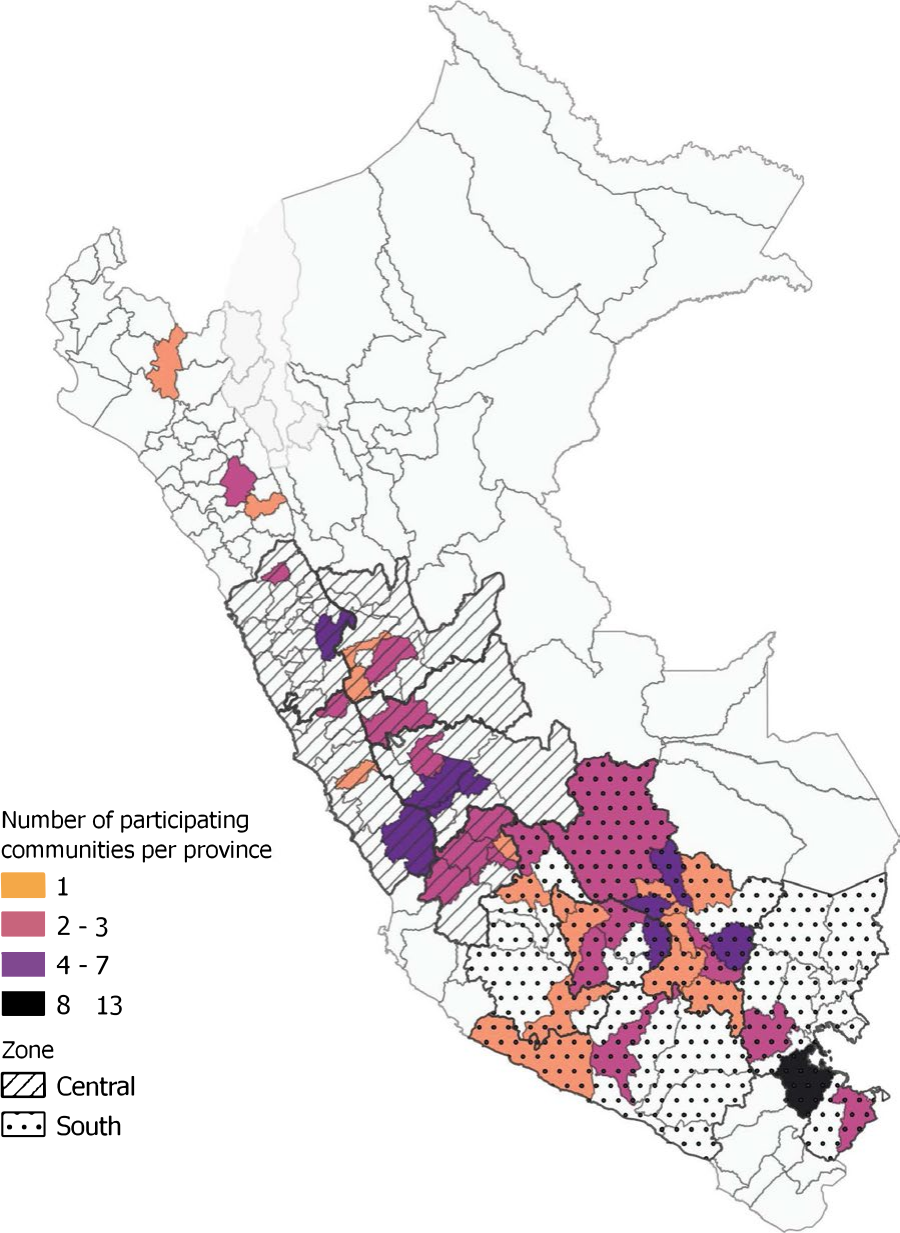
Map indicating the number of communities per province which participated in CIP’s repatriation program from 1996 to 2020, based on CIP (2021a)

Between 1997 and 2020, 14,950 landrace samples,^1^ including 1519 unique landrace cultivars, were distributed to 135 communities in the Peruvian Andes (CIP, 2021a). These distributions represent over 50% of the active accessions that were originally sourced from Peru (CIP, 2021a).^2^ Most landrace samples were distributed to the department of Cusco (7304), followed by Puno (1409), Ancash (1311), and Huancavelica (1205). Since the start of the program, each community received on average 111 samples, and most communities only participated once in the program. Within Peru, more accessions were distributed to the south and less to the north and center, as shown in Fig. 2. This figure indicates the number of participating communities per province and the central and southern zones defined for the duration model.

Over the years, the activities of the repatriation program have changed. Table 2 shows the number of samples and participating communities involved in the repatriation program over the years, displaying the upward trend in numbers and the sustained, continuous process. Due to its setup, the program is farmer-driven, and their interests have increased over time. We hypothesized that major contributors are farmer networks and word of mouth enriched by the close ties of the principal curator to the receiving communities or institutions, such as the Parque de la Papa (Potato Park) (R. Gómez, personal communication, 01 March 2021). As we could not single out all the driving factors using the survey data, we further speculate that demand for repatriated materials increased by changes in potato production and local loss of landraces due to the El Niño-Southern Oscillation and disruptions in potato production, loss of landraces and interruptions in seed systems caused by conflicts between the disturbances during the era of the guerrilla group Shining Path. Further farmers’ motivation might have changed due to their intrinsic preferences, market demands and globalization. Funding was also an influential factor, as there was no repatriation in 2009 due a lack of funding for the program. In 2020, the COVID-19 pandemic with at home confinements and other restrictions delayed the repatriations.

**Table 2:** Number of repatriated samples, and participating communities per year

Year	Number of repatriated samples	Number of participating communities
1997	488	2
1998	420	4
1999	535	7
2000	382	9
2001	191	4
2002	1356	8
2003	330	4
2004	290	7
2005	30	1
2006	1144	8
2007	156	3
2008	412	7
2009	0	0
2010	474	6
2011	402	1
2012	344	3
2013	725	7
2014	789	5
2015	1445	11
2016	1855	18
2017	542	6
2018	398	7
2019	1827	21
2020	415	5

The landrace accessions that have most frequently been repatriated (Table 3) are accessions with a wider geographical distribution. Even though the repatriation material was not selected based on any criteria other than availability and accession origin (meaning place where material was collected), most of the repatriated potatoes tended to have the following morphological characteristics: oblong, without unusual tuber shapes, slightly deep or deep eyes, cream as the predominant tuber flesh color without a secondary color, purple or violet tuber skin with intermediate to high intensity, and a wider range of secondary colors.

**Table 3:** Top ten most repatriated landrace accessions (CIP 2021a). Taxonomic designations are those from Hawkes (1990)

Accession number	DOI	Species or subspecies of Solanum	Number of repatriated samples
CIP 702013	10.18730/99TN	*Chaucha*	64
CIP 707136	10.18730/CS9U	*Chaucha*	62
CIP 700485	10.18730/8XZC	*Tuberosum andigenum*	51
CIP 701515	10.18730/960A	*Tuberosum andigenum*	49
CIP 703181	10.18730/9HVK	*Tuberosum andigenum*	47
CIP 703932	10.18730/A3Y6	*Tuberosum andigenum*	45
CIP 702037	10.18730/9A1W	*Tuberosum andigenum*	45
CIP 702961	10.18730/9H2Z	*Stenotomum goniocalyx*	44
CIP 700863	10.18730/9197	*Tuberosum andigenum*	43
CIP 700790	10.18730/90NR	*Tuberosum andigenum*	42

The genebank repatriation survey data also included the reasons why communities participated in the program. Most communities wanted to participate to restore their potato diversity and productivity (82% of all communities). The other justifications focused on potato diversity recovery (16%) and mitigation of climate change and natural disasters (2%). The mention of climate change shows that it is a push and pull factor for increasing potato diversity. Several communities mentioned that they lost some landraces due to unprecedented weather extremes (pull factor), and others noted that the repatriated material is needed for adaptation (push factor).

### Duration model: analyzing the survival of repatriated landraces

Over the program duration (1997–2018), 57% of all farming households included in the duration model ceased growing the repatriated landraces, 32% continued to plant the repatriated material at the time when the survey was conducted in August and September 2018, and for 11% no status could be retrieved. The longest survival time of local bundles of repatriated landraces was 20 years, the shortest was 1 year.

**Fig. 3:**
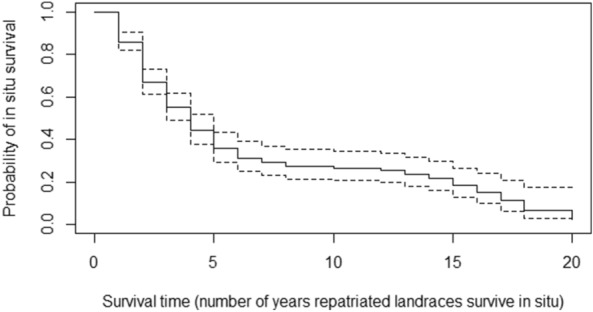
Kaplan–Meier survival curve displaying the in situ survival probability of the repatriated material at each successive year after the repatriations. The dashed line shows the 95% confidence interval of the estimation

The Kaplan–Meier curve (Fig. 3) shows the probability that the repatriated landraces survive in situ, i.e., that farmers continued to maintain them in their fields at a given point in time after receiving the repatriated material. Table 4 shows the probability of survival for each year after a household received repatriated material. It also shows how many households still maintained the repatriated landraces and did not abandon them, how many households abandoned repatriated materials, and how many censored households existed at each point in time. The probability of survival decreased steeply in the first few years after receipt of the material, indicating that most households abandoned the repatriated material during the first 4 years. As shown in Fig. 3, the curve declines at a slower rate and reaches a plateau until year 15. This indicates that for about 10 years, the survival probability stays in a relatively narrow range between 36% (year 5) and 18% (year 15). Afterwards, the curve drops again, and in year 20, the survival probability is only 3%.

**Table 4:** Results of the Kaplan–Meier survival analysis

Year after receiving repatriated material	Number of households			Survival probability
	Maintaining the material	Abandoning the material	Censored	
1	256	36	7	0.86
2	213	47	37	0.67
3	129	23	15	0.55
4	91	18	5	0.44
5	68	13	7	0.36
6	48	6	0	0.31
7	42	3	1	0.29
8	38	2	3	0.27
9	33	0	1	0.27
10	32	1	3	0.27
12	28	1	1	0.26
13	26	2	0	0.24
14	24	2	3	0.22
15	19	3	4	0.18
16	12	2	2	0.15
17	8	2	1	0.11
18	5	2	0	0.07
19	3	0	1	0.07
20	2	1	1	0.03

The second part of the duration model is the Cox proportional hazard analysis, which quantified the effect of household and regional characteristics on the survival time of the repatriated materials and revealed the statistically significant determinants of survival. Holding other covariates constant, the likelihood that a household abandons the repatriated material increased when the person in charge of the plots with repatriated material was male. The HR value of 1.71 indicated that a male plot manager increased the likelihood of abandonment 71% in comparison to a female in charge of the plots. This effect is statistically significant, and hence, is an important factor to predict survival. Farmers under 30 years of age were also significantly more likely to abandon the repatriated materials than older adults. On the contrary, the likelihood of abandonment decreased by a factor of 0.76 or 24% when the person managing the plots with repatriated material was over 60 years old. Nevertheless, the effect of these two age variables was not statistically significant. A higher level of education, larger labor force, and location in the southern geographical zone, had a statistically significant effect on survival times, indicating a lower risk of abandonment and hence implying longer survival times of repatriated landraces. The covariates household wealth and food insecurity in the district also had positive, but non-significant, effect on survival times.

The overall model was robust and statistically significant, based on tests for multicollinearity, the Weibull distribution, the proportional hazard assumption and influential observations.

### Benefits induced by the program and reasons for landrace abandonment

Most farmers (56%) noted nutritional improvement as the most important benefit induced by the program. These improvements were achieved through increased food consumption, food security, health, and an increased availability of landraces for traditional processing, such as “chuño” (freeze-dried potato). In the survey, 31% of participants reported nutritional and economic benefits, while only 13% reported pure economic benefits—related to improved possibilities to sell their product at local markets.

Culinary diversity was increased by the repatriation program. The surveyed farmers reported that they experienced an increase in the number of distinct flavors and textures. Unique flavors and textures were defined by different levels of sweet and bitter and a watery, starchy, or chewy texture. This increase in culinary diversity was also confirmed when female household members and those respondents who were mainly responsible for cooking were interviewed separately. Women reported an average of eight new combinations of flavors and textures brought to their kitchens by the repatriated material. Of the interviewed women, 72% felt healthier from eating landraces, and 81% acknowledged that their family or community benefitted from the program.

Of all the surveyed farmers, 77% said that the program helped them to recover lost landraces. They also perceived a union of traditional farming and scientific knowledge, and they enjoyed improved reputations from growing repatriated landraces.

The changes were not clear-cut regarding the effects of on-farm productivity. Of all the surveyed farmers, 57% stated that yield increases were induced by the program, but also a quarter of them revealed that these were not realized, and the remainder did not answer this question. Meanwhile, 41% of the respondents noted with the repatriated landraces an overall increase in total potato production, irrespective of the area sown. But 38% of the farmers did not notice a production increase, and again, the remainder did not provide an answer. Further, many respondents (44%) observed that they sold less outputs to the markets, as most repatriated material was used for home consumption, but 31% of them confirmed they had more surplus for the markets. The respondents were also divided about whether the program generated more income from market sales, and about the same number of interviewees responded yes and no.

Of the overall participants, 105 farmers (or 65%) stated that the main reason why they stopped cultivating the repatriated material was production loss due to abiotic stresses, especially loss from frost and hail (n = 33), which affected their production areas during extreme cold conditions. Other reasons for abandoning cultivation were biotic stresses, insufficient labor force or knowledge by the farming household, and lack of planting material.

## Discussion

Since 1997, CIP’s repatriation program has contributed to re-diversifying Andean potato production systems while creating nutritional, economic, and traditional benefits to the farmers. This re-diversification was enabled by a dynamic model of conservation, involving the CIP genebank and Andean communities. Currently, 135 Peruvian Andean communities received 14,950 landrace samples that comprise 1519 unique landrace cultivars.

The survival analysis showed that, on farms, much of the repatriated material did not survive the first 4 years, but later, the survival rate stayed relatively constant for 10 years, ranging between 36% in year 5 and 18% in year 15 (Fig. 3). This means that once the challenges during the first few years of repatriation are overcome and farmers have more experience with the redistributed material, the likelihood of survival stabilizes. The difficulties included factors such as lack of knowledge, time, labor force, or planting materials. The study found no evidence that the farmers discarded varieties because they did not value them. Meng (1997) and Meng et al. (1998) stated that farmers must have an intrinsic or underlying preference for landrace diversity if a landrace conservation program is to be successful. This preference is a prerequisite for diversity cultivation and conservation. Other reasons for diversity conservation are the maximization of benefits such as high yield levels and plant health. The cultural and historical ties of Andean smallholder communities to the potato crop are strong (de Haan 2006), as the Andes are the place of origin for the potato crop. Nevertheless, cultural ties are weakening, in particular in the younger generations, due to urbanization and a changing labor market as well as changing dietary preferences (Cavagnoud and Aramburú 2019).

The factors that increased the likelihood of long-term maintenance of the repatriated material on farms (Table 5) are in line with the large body of literature that has analyzed on-farm (potato) diversity and the factors that contribute to landrace cultivation. The characteristics of the plot manager, who is mainly responsible for the plots where the repatriated material is grown, largely influenced survival times. Our finding is that, in comparison to females in charge of the plots with repatriated material, male plot managers were more likely to abandon the repatriated landraces earlier. In the literature, a shared custodianship is described, as both female and male household members influence potato diversity, even though they are responsible for different tasks (de Haan 2006; Gruberg et al. 2013). Our gender variable cannot provide information on such a shared custodianship, as the survey only asked for the gender of the person who is mainly responsible for the potato plots with repatriated material. In Andean smallholder communities, women usually conduct the tuber selection after harvest, where they decide which tubers are best used for home consumption, sale, seed potatoes, and traditional methods of preservation. It is also considered their responsibility to safeguard the seeds for the next season (de Haan 2006). These tasks and the high use value of potato diversity increases the chances for their long-term maintenance.

**Table 5:** Results of the multivariate Cox regression model

Variable	HR
Gender (Male plot manager, reference: female plot manager)	1,71^*^
Age 60 + (Age of plot manager > 60 years, reference: age group 30 to 60 years)	0,76
Age below 29 (Age of plot manager < 29 years, reference: age group 30 to 60 years)	1,63
Education	0,80^*^
Labor force	0,96^*^
Wealth	0,92
Food insecurity	0,75
Zone South (Center is reference)	0,47^***^

Significance codes: *** p-value < 0.001; * p-value < 0.05

We found that older farmers were more likely to maintain repatriated landraces, nevertheless the variables’ effects are non-significant. Older farmers usually have more experience with potato production and stronger traditional ties to the crop and its production and likely place more value on conservation of the potato landraces. These factors are considered to positively influence farming household’s landrace diversity and maintenance (Cromwell and van Oosterhout 2000; Kruzich and Meng 2006; Perrault-Archambault 2007). Contrarily, some studies found that especially in the very heterogenous and high-altitude farming systems in the Andes, older-aged farmers were also found to have lower potato diversity. This is because maintaining potato diversity requires an extensive labor force, which can be reduced due to illnesses or other factors. For example, Gruberg et al. (2013) found that farmers between 25 and 55 years of age have the greatest variety portfolio. We hypothesize that our finding was influenced by the pronounced rural exodus of this age group due to migration (Cavagnoud and Aramburú 2019) and the strong traditional connections of older age groups to the repatriated material. Further evidence suggests that the presence of elderly farmers, and not necessarily their active conservation, enhances diversity (Negri 2003). Our model also includes the covariate labor supply, which shows how many people work on the potato plots of a household.

The labor force of households is determined by the number of its members who participate in the potato production, and it can be reduced by off-farm work and migration (Hellin and Higman 2005; Winters et al. 2006; Gruberg et al. 2013). In the Andes, potato production is very labor intensive, as it is no (purchased) input farming and studies on potato diversity of Andean farming households found that an increased number of available laborers enhances its potato portfolio (Zimmerer 1991; de Haan 2009; Arce et al. 2019). Also, in our model, the effect of internal and external labor force was statistically significant.

Another important variable determining a household’s human capital resources is education. Our results showed that higher education levels increased survival times. In context with the other results, we think that more education particularly helps the repatriated material enhanced diversity to survive during the first critical years, as educated farmers face fewer challenges regarding access to information such as information on the repatriation program, extension services, and cultivation tips.

Wealth had a small and non-significant effect, according to the model results. Literature suggests inconsistent effects of wealth to agrobiodiversity. On one hand, higher potato diversity levels might prevail among poorer farmers, as they need it for their sustenance and risk management strategy. On the other hand, less well-off farmers might not have the possibilities to manage great potato diversity due to labor, time, and knowledge disadvantages. Several authors have previously argued that custodian farmers are among the “better-offs” (Zimmerer 1996; Cromwell and van Oosterhout 2000; Smale 2006; Winters et al. 2006; Perrault-Archambault 2007; Lüttringhaus et al. 2016). In contrast, wealthier farmers also profit most from political changes in the Andes (Zimmerer 1996), and they can maintain high potato diversity levels as they possess more land, they can afford to remunerate additional workers, and they can buy seeds. Again, these are factors that increase the intrinsic motivation of farmers for diversity, and hence, lead to a higher likelihood of maintaining the material. Gruberg et al. (2013) found that middle to middle-low-income farmers cultivate the greatest number of potato varieties. They have more land than the poorest families but not as much income as well-to-do families. Hence, they cannot afford a great variety of potato substitutes and must cultivate a high level of potato diversity to assure food security.

Similarly, our duration analysis showed that the likelihood of survival increased with higher average levels of food insecurity at the district level where the household is located, but the effect was not significant. In the study area, potato landraces are usually planted as “chaqru”, which is a mixture of landraces for risk management (de Haan 2009). In combination with the heterogenous farming landscape stretched over numerous altitudinal belts (Winters et al. 2006), chaqru allows farmers to harvest different plots over the year and to stretch fresh potato harvests over longer periods of the year (Clawson 1985; Smithson and Lenné 1996).

The location of the households determines survival times. Our analysis showed that the material survived longer in the south of Peru. The southern region is an important center of potato landrace diversity and includes the department of Cuzco, where the tourism sector is strong due to the world heritage site of Machu Picchu. Also, the Potato Park was established in this region, which is globally the largest in situ conservation area for potato diversity (Hall 2019). The higher survival times in the south might be also influenced by the longstanding ties between some of the communities and CIP’s principal potato curator, who fostered and built strong relationships with the farmers in these areas. In the other investigated zone, the center of Peru, survival times are lower than in the south. In the center, there could be more potato substitutes, as agriculture there is less potato-centered. The importance of location and the underlying preference for landrace diversity are crucial for diversity (Meng 1997; Arce et al. 2019).

The benefit and change analysis revealed that the repatriation program contributed to improving the livelihoods and food security of participating farmers. The program increased family nutrition by having a positive impact on food quantity, dry matter content, yield, production, etc. Further, the possibility to use some of the repatriated material for traditional processing techniques, such as chuño (freeze-dried potato for longer-term storage), supports food security, as it provides readily available food when the fresh tubers are depleted. Also, the culinary diversity was increased by providing landraces with new tastes and textures. Such an increased diversity is very welcomed by farmers whose diet is dominated by potatoes and therefore it is a “luxury” for them to choose from a wider set of potato tastes, textures, shapes, and colors (de Haan 2009, p. 179). The women interviewed benefited especially from this diversity, and they perceived a significant health benefit for their families.

Economic and cultural benefits, such as a union of traditional and scientific knowledge, were noted by survey respondents, suggesting that farmers perceived and implemented the dynamic model of conservation in practice. These findings underscored that farmers recognized the private economic value of repatriated landraces. Devaux et al. (2020) showed that there is evidence of an emerging value of potato landraces and the benefits they induce, partly because of new culinary trends. These values and incentives are crucial to understand the in situ maintenance of landraces (Meng et al. 1998; Smale et al. 2001). Further, the analysis revealed that the process of repatriation, including the multiplication of material by farmers, created a sense of ownership of the program, which can facilitate the long-term benefits of the program and its future upscaling activities. Ownership can be also supported by famers’ perception of benefits for women. Our findings indicated that not only was the main objective of the program (recovery of lost landraces) (Huaman et al. 1999; Ellis et al. 2020) achieved, but that farmers’ perceived benefits were varied beyond this primary aim. In line with our findings, other analyses of on-farm maintenance projects of crop diversity in the Andes showed that these programs served a dual purpose by fostering in situ conservation while creating positive livelihood outcomes (Bellon et al. 2015).

The need to support farmers’ adaptation capacity was underscored by the finding that the main reasons for a loss of the repatriated material are abiotic and biotic stressors. Climatic and environmental conditions have been changing the Andean region (Arce et al. 2019; Hock et al. 2019), and hence, plots or altitudinal belts where specific landrace cultivars grew before they were lost might not be suitable anymore, as farmers have needed to move upslope to deal with the changing conditions. Also, it is possible that the adverse conditions that lead to the loss of plots in the first place persist, and hence, they contribute again to the loss of landraces. Future monitoring should integrate these aspects to detect the main driving factors of loss.

Many farmers also stated that they lacked enough farm laborers to maintain the repatriated material. The duration model confirmed that a smaller labor force in the household increased the likelihood of abandoning the repatriated landrace. Further, a lack of knowledge was mentioned as a limiting factor for maintenance of repatriated material. Here, the gap between generations becomes evident: younger farmers often prefer to seek education and labor opportunities in urban areas, which inhibits an intergenerational exchange to convey the knowledge on potato production. Other studies have also found that a shift from one generation to the next led to landrace loss (McLean-Rodríguez et al. 2019). This risk could be decreased by intergenerational apprenticeships between older and younger farmer generations.

It is important to note that the reasons for loss of repatriated material are highly interconnected and are often location and landrace specific (McLean-Rodríguez et al. 2019). Ideally, solutions require comprehensive local monitoring or mentoring of farmers to act as a connection between the participating farmers and CIP (in cases where advice is needed).

Another reason for abandonment was related to the organization and management of the program. This underlined that any repatriation program requires a solid institutional structure and long-term institutional commitment to multiply the repatriated material in sufficient quantity so that all willing farmers receive material and ensure the future success of the program. In two communities, problems occurred during the communal multiplication process of the repatriated material. Community members did not organize well and hence the material was not sufficient for all farmers who wanted to participate. Some farmers perceived that the distribution of multiplied material was unfair, and discontent was created. After these experiences, the selection process for participating communities was adjusted accordingly.

Our study is the first comprehensive description and evaluation of CIP’s 20-year-old repatriation activities and the first analysis to apply a duration model to study the long-term survival of landraces on household farms. The analyses were based on a solid set of data: passport data on the repatriated accessions, a household survey on the changes induced by the repatriated material, and interviews with involved CIP staff. Thus far, the repatriation program is part of the routine genebank work at CIP and has not been institutionalized as an individual project or received specific programmatic support. Such a transformation could enhance the repatriation work and help to generate further evidence of impacts to smallholder farmers in Peru and beyond.

Most communities who received repatriations, only participated once in the program (93%). Only ten communities received more than one repatriation, the maximum of repatriations to one community was four. Communities could participate as often as they wanted, however this program is community-driven, and it takes active effort for the communities to organize and request material from CIP. CIP makes it as easy as possible but often initial efforts to organize the repatriations are initiated by NGOs working with the communities and in the absence of the NGOs initial organizational efforts, the communities lack the resources, drive or information for subsequent requests.

The household data set was very comprehensive and detailed, yet there were some limitations. A few well-established variables for duration analyses, such as the potato plot or farm size, were not included in the questionnaire, and therefore, could not be integrated in our model. We had a limited number of quantitative and continuous variables per household and used recall data for the duration model, which means that farmers might have incorrectly recalled how long they have been planting repatriated material. Therefore, if such factors are to be included in the future, monitoring is advised and should be done in close collaboration with experts in survey design and socioeconomics. Another limitation was that the specifics of the material a household received was not documented. Based on the survey we know what material was received by the community. As we cannot assume that all participating farmers in one community received planting material for all landraces repatriated to the community, we could not analyze how survival times differed according to specific landraces or their traits. Such information could be valuable for future identification of potentially valuable landraces. Nevertheless, it is again important to note that the risk-minimizing planting strategy, chaqru, was most valuable due its overall trait spectrum of landraces. Further, since we had no information on farmer-to-farmer distribution of the repatriated material, we could not deduce the survivability of repatriated material beyond an individual household to the community scale or beyond the individual community to multiple communities. This last element is crucial for conservation strategies. Such seed exchange is generally common amongst Andean farmers to renew or replace their potato portfolios (de Haan 2009).

## Conclusions

Our analyses have led to the first comprehensive description of the potato repatriation activities conducted by the CIP genebank. Further, we identified household and community factors that foster long-term maintenance of the repatriated landraces and the perceived benefits of repatriation for participating farmers and their communities.

Due to climatic and demographic changes, it is vital to revive and maintain the rich agricultural systems in the Andes and allow genetic resources to naturally adapt and evolve. Overall, a third of the interviewed participating farmers still conserved the repatriated material. Significant factors in the duration analysis and reasons cited by farmers for loss of the repatriated material were diverse and ranged from a shortage in labor force and knowledge, abiotic and biotic stressors, to community disorganization. Most participating farmers confirmed that they perceived a multitude of benefits induced by the repatriation, even if they stopped planting the material. The most mentioned benefits were the recovery of lost landraces and nutritional benefits. The program has fulfilled its main objective to return lost landraces by distributing healthy planting material, increasing farmers’ food security as well as the intraspecific potato diversity they manage. These results show that the program goals are very complex and highly interlinked with other factors that influence farmers’ livelihood. One factor is climate change, which is altering growing conditions at an unprecedented speed and intensity, including through abiotic and biotic stressors. Also, environmental degradation, urbanization, and changes in dietary and lifestyle preferences have intensified the changes in farming communities in the Andes.

We provide evidence that a continuation or upscaling of CIP’s repatriation activities could generate benefits to food insecure regions and is a way to improve the livelihoods of many farming communities that depend on potato production for sustenance and traditions. Due to the large number of smallholder farmer communities in Peru and other Andean countries, the upscaling potential of the repatriation activities would be large and would require substantial investment. Up to and including the year 2020, 135 different communities have participated in the repatriation program, but there are about 6000 smallholder communities in Peru (Diez-Hurtado 2011; Pajuelo Teves 2019). This means that 0.02% of the communities have participated so far and that many more could benefit from the program by receiving clean and diverse planting material. To continue the repatriation work, support and funding must be secured.

## Data Availability

The datasets used and/or analyzed during the current study are available from the corresponding author on reasonable request.

## Abbreviations

CGIAR: Consultative Group of International Agricultural Research
CIP: International Potato Center
HR: Hazard ratio
m.a.s.l: Meters above sea level
ITPGRFA: International Treaty on Plant Genetic Resources for Food and Agriculture
SMTA: Standard Material Transfer Agreement

## Footnotes

1 Here sample refers to a set or certain number of tubers of one unique landrace cultivar. Each unique landrace cultivar corresponds to one accession in the CIP genebank.

2 As of February 2021, the CIP genebank has 7466 cultivated and wild potato accessions (CIP 2021b). Of these, 4870 are cultivated and 2596 are wild. The number of active landraces in the CIP collection is 4467, and of these, 3427 are currently available for distribution. Over time, the CIP genebank has repatriated 1519 of all landrace accessions. These numbers also include landraces form other countries of origin. For Peru, the landrace collection currently holds 2854 active accessions, of which 2114 are available for distribution. And, over time, 1498 have been repatriated by the repatriation program analyzed here (CIP 2021b). These numbers are dynamic because of the continuous genebank operations.
